# Rapid Detection of the Varicella-Zoster Virus Using a Recombinase-Aided Amplification-Lateral Flow System

**DOI:** 10.3390/diagnostics12122957

**Published:** 2022-11-25

**Authors:** Kathrina Mae Bienes, Lingjing Mao, Benjamin Selekon, Ella Gonofio, Emmanuel Nakoune, Gary Wong, Nicolas Berthet

**Affiliations:** 1 Unit of Discovery and Molecular Characterization of Pathogens, Center for Microbes, Development and Health, Institut Pasteur of Shanghai, Chinese Academy of Sciences, Shanghai 200031, China; 2 University of Chinese Academy of Sciences, Beijing 100049, China; 3 Institut Pasteur of Bangui, Bangui, Central African Republic; 4 Viral Hemorrhagic Fevers Research Unit, CAS Key Laboratory of Molecular Virology and Immunology, Institut Pasteur of Shanghai, Chinese Academy of Sciences, Shanghai 200031, China; 5 Cellule d’Intervention Biologique d’Urgence, Unité Environnement et Risque Infectieux, Institut Pasteur, 75724 Paris, France

**Keywords:** varicella, chickenpox, monkeypox, smallpox, RAA, lateral flow, diagnostics, point-of-care-test

## Abstract

Varicella-zoster virus (VZV) is the etiological agent of varicella (chickenpox) and herpes zoster (shingles). VZV infections are ubiquitous and highly contagious, and diagnosis is mostly based on the assessment of signs and symptoms. However, monkeypox, an emerging infectious disease caused by the monkeypox virus (MPXV), has clinical manifestations that are similar to those of VZV infections. With the recent monkeypox outbreak in non-endemic regions, VZV infections are likely to be misdiagnosed in the absence of laboratory testing. Considering the lack of accessible diagnostic tests that discriminate VZV from MPXV or other poxviruses, a handy and affordable detection system for VZV is crucial for rapid differential diagnosis. Here, we developed a new detection method for VZV using recombinase-aided amplification technology, combined with the lateral flow system (RAA-LF). Given the prevalence of VZV worldwide, this method can be applied not only to distinguish VZV from other viruses causing rash, but also to foster early detection, contributing substantially to disease control.

## 1. Introduction

Varicella-zoster virus (VZV), also known as human herpesvirus 3 (HHV-3), is an α-herpesvirus that is the etiological agent of varicella (chickenpox) as a primary infection and herpes zoster (shingles) as a recurring infection [1,2,3,4,5]. VZV is specific to humans [4,6], and is extremely contagious via airborne transmission [3,4,6]. Most VZV infections result in varicella, causing fever and a generalized pruritic rash, which usually occurs during childhood [1,3,6]. Sharing a common feature of herpesviruses, VZV undergoes latency and may reactivate. Generally occurring in adults, VZV reactivation causes herpes zoster (HZ), producing a localized, painful rash [1,2,4,6]. Both varicella and zoster are prevalent worldwide [1,3], and can be serious, especially in children and adults with weakened immune systems [1,4,6].

Varicella is a rather neglected illness that has not been given much attention. To some extent, it is considered more of a nuisance than an actual disease [5,7]. Although VZV does not belong to the family of poxviruses, varicella is commonly called chickenpox, likely due to its resemblance to variola (smallpox) virus infections [8], albeit with milder symptoms [7]. Smallpox has been declared eradicated since 1980 by the World Health Organization, but other orthopoxviruses still pose a substantial threat to public health [9]. Monkeypox virus (MPXV), the next-of-kin to variola virus (VARV), has emerged as the most important orthopoxvirus in the post-smallpox era [8,10,11,12,13]. MPXV is the causative agent of monkeypox, a rare zoonosis endemic to West and Central Africa [8,10,14]. However, since early May 2022, an outbreak of monkeypox has been ongoing in non-endemic regions, such as Europe and the Americas [15,16].

The clinical manifestations of varicella are nearly identical to those of monkeypox, making them generally indistinguishable without laboratory testing [11,12,17,18]. Varicella and monkeypox coinfection has also previously been reported [19,20]. Hence, confusion in the diagnosis of varicella and monkeypox is common in areas where the viruses coexist, particularly in Central Africa [8,12,18]. Laboratory diagnostics are essential for the identification and surveillance of these diseases [11,18], and there are several laboratory tests, but they are not always available due to limited diagnostic resources in developing regions [2,3,5]. The “gold standard” for the diagnosis of VZV infections was once virus isolation, but this is time-consuming, costly, and not readily accessible [2,8,15]. This method has now been replaced by polymerase chain reaction (PCR) and direct fluorescence assays (DFA) as the methods of choice [1,11,12]. PCR, including quantitative PCR (qPCR), is considered the most sensitive and reliable testing method for VZV to date [1,11]. However, it requires expensive reagents and equipment, as well as a high degree of skill and laboratory experience [3,11].

Cases of human monkeypox are more common in Africa, where surveillance can be more challenging due to poor infrastructure and lack of healthcare facilities [11,21]. The Center for Disease Control and Prevention (CDC) has recommended a number of laboratory procedures to confirm monkeypox infections. However, these procedures are mostly based on nucleic acid amplification testing (NAAT) via PCR and/or sequencing [22,23,24,25,26,27,28]. Before its emergence in non-endemic regions in 2022, monkeypox was a neglected tropical illness that most people were not even aware of. Thus, research to develop more efficient detection methods for MPXV has lagged until recently. Development of detection tests (e.g., loop-mediated isothermal amplification, LAMP; recombinase polymerase amplification, RPA; restriction length fragment polymorphism, RLFP) for MPXV is becoming a subject of active research due to the recent outbreak [29,30,31], but that is not the case for VZV. However, since clinical distinction among varicella, monkeypox, and other rash illnesses is almost impossible in the absence of a diagnostic test, new tests are needed for a more precise and rapid diagnosis [11,18].

Here, we developed a rapid detection method for VZV using recombinase-aided amplification-lateral flow (RAA-LF) technology. The gene of interest is the VZV open reading frame 63 (ORF63), which encodes an immediate early protein (IE63) that is synthesized during lytic infection [32]. IE63 is also expressed during the latent phase, indicating a critical role in the maintenance and establishment of virus latency [33,34,35]. The whole process, from the RAA reaction to the visualization of the band on the LF dipstick, can be completed in less than 30 min.

## 2. Materials and Methods

### 2.1. Sample Description and DNA Extraction

The samples used in this study were collected during investigations carried out by the Institut Pasteur of Bangui (IPB) in the Central African Republic (CAR), when suspected cases of MPXV was reported by the health authorities. On each occasion, IPB conducted field missions to collect biological samples (crust, serum, or pus) and epidemiological information for each reported case. From the DNA extracted from these samples, IPB investigated the etiological cause by determining whether the agent was MPXV or VZV. When a suspected case of MPXV is reported, investigation for both MPXV and VZV is done systematically by IPB. The case is confirmed to be either MPXV or VZV, or occasionally, a co-infection of both viruses. Of the 20 samples, 15 were collected from crusts of lesions, and the other five were from pus. Detailed information on the samples is reported in Table 1.

The phenol-chloroform method was employed to isolate nucleic acid as described below. One milliliter of the extraction solution (ES) consisting of 100 mM EDTA (Invitrogen, Waltham, MA, USA), 200 mM NaCl (Invitrogen, Waltham, MA, USA), 50 mM Tris-HCI (pH 8.0) (Solarbio Life Sciences, Beijing, China), 0.5% SDS (Solarbio Life Sciences, Beijing, China), and 50 μg/mL RNase (Sigma-Aldrich, Burlington, MA, USA) was added to the sample, followed by the addition of 20 μL Proteinase K (100 μg/mL) (TIANGEN Biotech Co., Ltd., Beijing, China) and gentle mixing. The solution was incubated at 55 °C for at least 2 h with occasional mixing. An equal volume of phenol (Solarbio Life Sciences, Beijing, China) was added to the solution, and was vortexed vigorously for 1 min. The tube was centrifuged at 9600× *g* for 5 min at 4 °C to separate the two phases. The aqueous (top) phase was then transferred to a new tube, and an equal volume of phenol:chloroform:isoamyl alcohol (25:24:1) (Solarbio Life Sciences, Beijing, China; Sinopharm Chemical Reagent Co., Ltd., Shanghai, China; Richjoint Chemical Reagents Co., Ltd., Shanghai, China) was added to the aqueous phase. The tube was vortexed for about 1 min, and was centrifuged at 12,000 rpm for 5 min at 4 °C to separate the two phases. The aqueous phase was again transferred to a new tube, and an equal volume of chloroform:isoamyl alcohol (24:1) (Sinopharm Chemical Reagent Co., Ltd., Shanghai, China; Richjoint Chemical Reagents Co., Ltd., Shanghai, China), was added to the aqueous phase. The tube was mixed, and was centrifuged at 9600× *g* for 5 min at 4 °C to separate the two phases. The aqueous phase was transferred again to a new tube. Then, 14 µL of 5M NaCl with a final concentration of 0.14 mol/l was added to the mixture, and the solution was mixed thoroughly. Two volumes of absolute ethanol (Sinopharm Chemical Reagent Co., Ltd., Shanghai, China) were added to the aqueous phase, and the solution was mixed gently. The tube was kept at −20 °C for 30 min, and was centrifuged at 9600× *g* for 5 min. The DNA pellet was washed with 70% ethanol twice to decrease the residual salt, and was air-dried at 37 °C to let the ethanol evaporate. The ethanol-free DNA was then dissolved in 50 μL nuclease-free water (Invitrogen, Waltham, MA, USA).

### 2.2. Primer and Probe Design

Primers and probes targeting the ORF63 gene of VZV (GenBank: NC_001348.1:110581-111431) were manually selected for qPCR and RAA-LF assays (Table 2). RAA primers and probes were designed in accordance with the TwistAmp^®^ nfo kit assay design manual guidelines (www.twistdx.co.uk) (accessed on 26 July 2022). To assess the feasibility of amplification of the RAA primers, conventional PCR was performed in duplicate. The 50 µL PCR reaction consisting of 25 µL DreamTaq™ Hot Start Green PCR Master Mix (Thermo Scientific™, Waltham, MA, USA), 1 µL forward primer (10 µM), 1 µL reverse primer (10 µM), 5 µL DNA template, and 18 µL nuclease-free water was run using the MiniAmp Thermal Cycler (Applied Biosystems™, Waltham, MA, USA) under the following conditions: initial denaturation at 95 °C for 1 min, 25 cycles of 95 °C for 30 s (denaturation), 62 °C for 30 s (annealing), and 72 °C for 1 min (extension), and final extension at 72 °C for 5 min. Amplification was verified by agarose gel electrophoresis, and the gel was visualized under UV light using the Tanon 4200SF gel imaging system (Tanon Science & Technology Co., Ltd., Shanghai, China). The corresponding sequences of all primers and probes (Sangon Biotech Co., Ltd., Shanghai, China) used in this study are listed in Table 2.

### 2.3. RealTime-PCR (qPCR)

The VZV qPCR detection was designed to determine the concentration of the VZV plasmid in different dilutions, as well as to quantify the viral load of the VZV samples. A total volume of 20 µL qPCR reaction was comprised of 10 µL NovoStart^®^ Probe qPCR SuperMix (Novoprotein Scientific, Inc., Suzhou, China), 1 µL qPCR forward primer (10 µM), 1 µL reverse primer (10 µM), 0.4 µL probe (10 µM), 6.6 µL nuclease-free water, and 1 µL DNA template. qPCR cycling parameters were set to 95 °C for 5 min, plus 40 cycles of 95 °C for 15 s and 60 °C for 1 min, and was run using the Applied Biosystems QuantStudio 1 Real-Time PCR System (Applied Biosystems™, Waltham, MA, USA).

### 2.4. Recombinase-Aided Amplification–Lateral Flow

To visually detect VZV on a dipstick, the RAA-LF assay was conducted using the RAA nfo kit ZC Bio-Sci & Tech Co., Ltd., Hangzhou, China) and the HybriDetect universal lateral flow assay kit (Milenia Biotec GmbH, Geissen, Germany). The RAA nfo reaction consisting of 25 µL Buffer A, 2 µL forward primer (2 µM), 2 µL biotinylated reverse primer (2 µM), 0.6 µL probe (2 µM), and 15.9 µL nuclease-free water, was added to one tube of RAA lyophilized powder. The reaction was mixed thoroughly, followed by the addition of 2 µL DNA (in the tube), and 2.5 µL Buffer B (on the lid), respectively. The resulting 50 µL reaction was mixed vigorously before incubating at 37 °C for 10 min in MiniAmp Thermal Cycler (Applied Biosystems™, Waltham, MA, USA). For the LF dipstick assay, 2 µL of the amplification product was diluted with 98 µL of HybriDetect Assay Buffer. For each sample to be analyzed, 100 µL of the same buffer was prepared in another tube. Ten microliters of the diluted amplified product was pipetted on to the sample application area of the dipstick, and the dipstick was immediately placed into the solution in an upright position for no more than 10 min. Amplification was confirmed by the appearance of the test band along with the control band on the dipstick.

### 2.5. Assay Sensitivity and Specificity

The sensitivity of the qPCR and RAA-LF methods was assessed using a recombinant puc57 plasmid with the ORF63 gene in full length (Sangon Biotech Co., Ltd., Shanghai, China) as the standard. The concentration of the undiluted plasmid was quantified using the Qubit 4 fluorometer (Invitrogen, Waltham, MA, USA). The value obtained was needed to calculate the corresponding copy number using the following equation:

Number of copies = [plasmid concentration (ng/µL) × (6.0221 × 10^23^ molecules/mole)] ÷ [total plasmid length (bp) × 660 g/mole × (1 × 10^9^ ng/g)]; where, total plasmid length = vector length (bp) + fragment length (bp)

The limit of detection (LOD) was verified by qPCR, in triplicate, using several dilutions of VZV plasmid solutions with known copy numbers. A standard curve was established using these dilutions. The LOD determination by RAA-LF was also conducted using the same plasmid dilutions. The specificity of the RAA-LF assay was determined using VZV, MPXV, and vaccinia virus (VACV) DNA as templates. The viral DNA of MPXV was provided by IPB. A culture of VACV (ATCC^®^ VR-2047™) was obtained from the Pathogen Discovery and Big Data Platform of the Institut Pasteur of Shanghai–Chinese Academy of Sciences (IPS-CAS). VACV was then cultivated in Vero cells prior to DNA extraction using the GeneJET Viral DNA and RNA Purification kit (Thermo Scientific™, Waltham, MA, USA) according to the manufacturer’s instructions.

## 3. Results

### 3.1. Sampling Information

**Figure 1:**
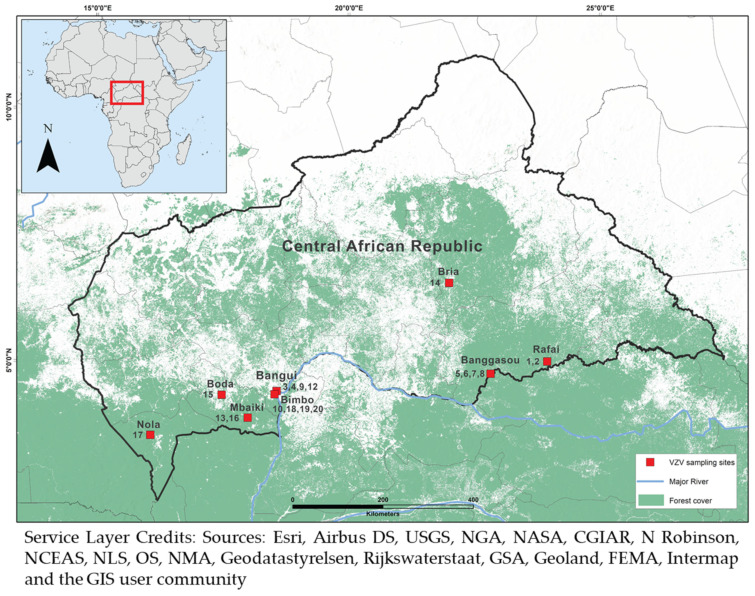
Sampling sites in the Central African Republic (CAR). Twenty VZV samples were collected from various locations in the CAR from 2018 to 2021.

A total of 20 samples was collected over a four-year period (2018–2021) from different cities in the CAR, including Rafaï, Bangassou, Mbaïki, Bria, Boda, Nola, Bimbo, and the capital city Bangui. The majority (14) of the samples was acquired from male patients, with five from female patients, and one of unspecified gender (Table 1). With respect to age, nine samples were obtained from adults over 18 years of age, seven from children under 18, and four from patients of unknown age (Table 1). Sampling sites were mapped using the ArcGIS^®^ software (Esri, Redlands, CA, USA) (Figure 1).

### 3.2. Primer Validation

Four sets of primers were manually designed for RAA-LF. The specificity of all primers and probes were confirmed using the NCBI BLAST tool to ensure the absence of cross-reactivity with related viral sequences. To validate the amplification feasibility of the primer pairs, conventional PCR assay and agarose gel electrophoresis were conducted. Results show that amplification was successful for all primer pairs, but a clearer band on the gel was produced by the first set of primers with an amplicon size of 227 bp (Figure S1). Primer set 1 was thus the primer pair of choice for the subsequent steps, and was used to derive the corresponding nfo probe. For the RAA nfo assay, the 5′ end of the chosen reverse primer (RAA-LF R1) was biotinylated (Table 2).

### 3.3. Assay Sensitivity and Specificity

**Figure 2:**
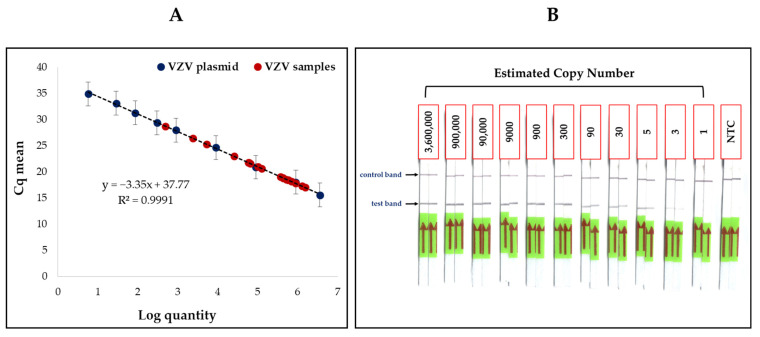
Limit of detection for VZV. qPCR and RAA-LF were conducted to determine the detection threshold for VZV with a recombinant plasmid. A standard curve was established using the mean threshold (Cq) values of the plasmid dilutions ((**A**), dark blue dots) obtained by qPCR, showing detection of VZV in samples with as little as 5 copies/µL. The mean Cq values of the VZV samples ((**A**), red dots) were also plotted on the standard curve against the VZV plasmid, showing a nearly linear regression line. The RAA-LF assay exhibited results similar to that of qPCR (**B**), with the test band present in samples with an estimated copy number of 5 copies/µL, and the no-template control (NTC) displaying only the control band. Both qPCR and RAA-LF are thus highly sensitive methods in detecting VZV.

A recombinant plasmid containing the full-length ORF63 gene of VZV with a copy number of approximately 3.60 × 10^9^ copies/µL was used to determine the detection threshold for of qPCR and RAA-LF. Plasmid dilutions with copy numbers ranging from 3.60 × 10^6^ to 5 copies/µL were initially used to determine the LOD by qPCR. A standard curve was also plotted using these dilutions (Figure 2A, dark blue dots), and was used to determine the copy number of the VZV samples. The curve gave an R^2^ value of 0.9991 and a slope (m) value of −3.35, suggesting a nearly linear trend and high PCR efficiency. The LOD for VZV was also determined using RAA-LF, in duplicate, with the same dilutions mentioned above for qPCR (Figure 2B). However, since intense bands were still observed even at a low concentration of 5 copies/µL, the RAA-LF assay was performed for two additional dilutions with an approximate copy number of 3 and 1 copies/µL, respectively. Slight test bands were observed in samples with 3 copies/µL, but was absent at 1 copies/µL concentration (Figure 2B). Figure 2 shows that both qPCR and RAA-LF were able to detect VZV at a very low concentration using the standard plasmid.

**Figure 3:**
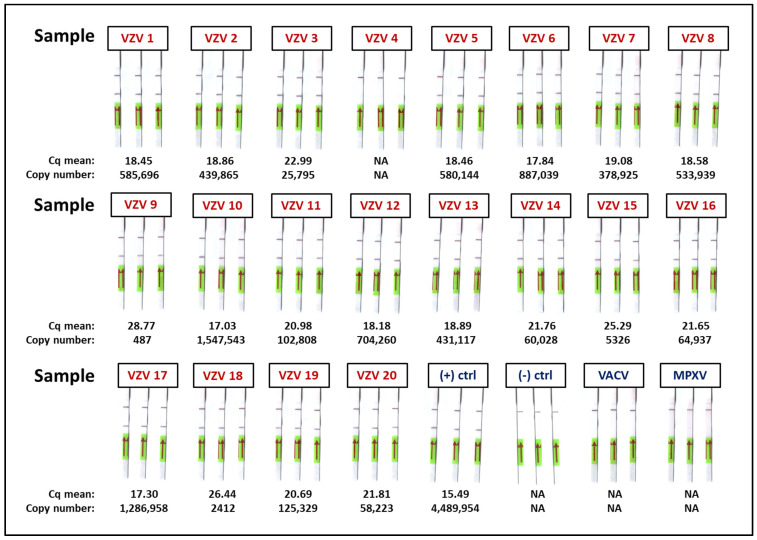
Detection of VZV in clinical samples. Twenty VZV samples were tested with qPCR, but one (sample 4) tested negative for VZV. The positive control used was the VZV plasmid with a concentration of about 3.62 × 10^6^ copies/µL, and nuclease-free water for the negative control. The specificity of RAA-LF was determined using the DNA of monkeypox virus (MPXV) and vaccinia virus (VACV), and both viruses tested negative for VZV. RAA-LF for VZV detection was also conducted, in triplicate, using the same clinical samples tested with qPCR. Nineteen samples, along with the positive control, tested positive for VZV by RAA-LF with the presence of the test band on the dipstick, while sample 4 only exhibited the control band, conforming to the results of the qPCR.

The specificity of RAA-LF was also verified using MPXV and VACV DNA as templates. The test was conducted in triplicate, and the viruses tested negative for VZV, suggesting that this test is highly specific to VZV (Figure 3).

### 3.4. VZV Detection in Clinical Samples with qPCR and RAA-LF

qPCR was performed in triplicate to confirm the presence of VZV in 20 samples. Nineteen of the 20 samples tested positive for VZV by qPCR, excluding sample 4, which tested negative. Sample 4 was collected from a patient with pustules that tested positive for VZV at IPB, but by the time this experiment was conducted at IPS-CAS, the sample was degraded. This negative result was verified after running another qPCR in duplicate. The corresponding mean threshold (Cq) value and copy number for each sample are presented in Figure 3. These results show that VZV was detected with copy numbers ranging from 487 copies/µL to 1.55 × 10^6^ copies/µL (samples 9 and 10, respectively). RAA-LF was also conducted, in triplicate, for the 20 clinical samples tested with qPCR. The presence of both the test band and the control band was observed, except for sample 4, which showed only the control band, in accordance with the qPCR results. The summary of the results is presented in Table 3.

## 4. Discussion

VZV is ubiquitous in nature, and laboratory confirmation is not routinely practiced to detect VZV infections. The samples used in this study were collected from the skin lesions of patients initially suspected to be infected with monkeypox, but turned out to be infected with VZV. Due to concurrent cases of VZV and MPXV, diagnosis based on clinical grounds alone is challenging and laboratory testing is necessary. The cases of VZV infections in the CAR were detected in the same cities and towns where monkeypox cases were also observed. These localities are close to remote forest areas where spillover events, including the enzootic monkeypox, occur [36,37,38] (Figure 1). Since outbreaks of VZV and MPXV infections have been reported simultaneously in the same regions, it is imperative to develop a rapid detection method not only for VZV, but also for MPXV, and potentially other poxviruses as well. With the current emergence of monkeypox as an infectious disease, proper clinical response for rash patients relies on a rapid detection test readily available to distinguish between VZV and MPXV during outbreaks.

Given its rarity, MPXV can be mistaken for VZV if it is diagnosed based entirely on clinical presentation, especially in remote areas where laboratory testing cannot be performed. Considering that diagnostic tools for both VZV and MPXV are limited, a rapid detection system for both viruses is necessary in case such situations arise. Isothermal nucleic acid amplification tests (iNAT), such as LAMP and RPA, have long been established for pathogen detection, and have been reported as good PCR alternatives [29,31]. However, one drawback of the LAMP assay is that it requires multiple sets of primers, in contrast to RAA/RPA, in which only a single pair of primers and a probe are necessary. A highly specific molecular detection system for MPXV has also been developed by our team using recombinase-based isothermal amplification techniques. Although orthopoxviruses are highly cross-reactive, three different RAA/RPA approaches (including visualization of the amplified product on a dipstick) have proven greatly successful in detecting MPXV in clinical samples [39].

**Figure 4:**
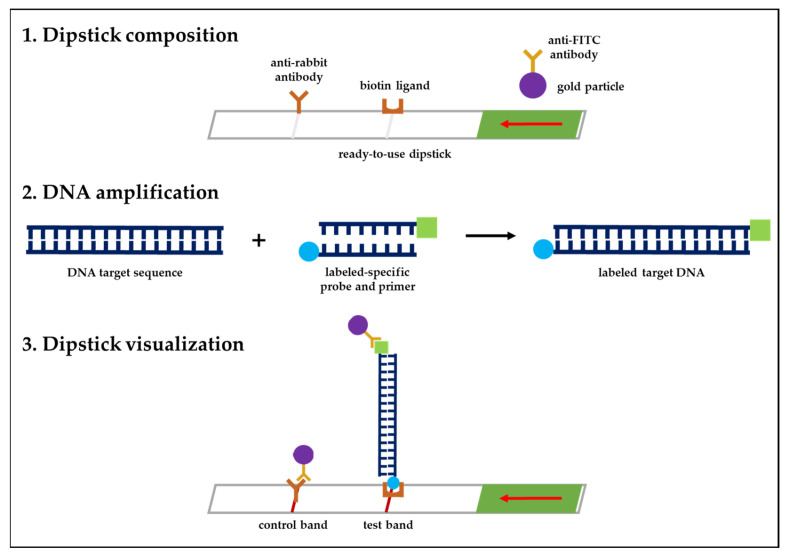
The lateral flow mechanism of the HybriDetect dipstick. (**1**) The sample application area of the dipstick is composed of gold-labeled FITC-specific antibodies. (**2**) Amplification of DNA by RAA nfo is performed using analyte detectors labeled with FITC (probe) and biotin (primer) that attach to the DNA target sequence. (**3**) The labeled analytes bind to the gold-labeled FITC-specific antibodies, travel to the membrane, and bind to immobilized biotin-ligand molecules while passing the test line, eventually generating a red-blue band. Unbound gold particles then migrate to the control line.

The RAA technique combined with the LF system was employed in this study to develop a detection method for VZV. As an enzymatic procedure, RAA is a brilliant scientific breakthrough that has already been successfully integrated in different detection strategies [40]. HybriDetect, on the other hand, is a ready-to-use test strip based on lateral flow technology using gold particles. The dipstick was designed to rapidly detect analytes, such as gene amplicons, labeled with FITC (probe) and biotin (primer). In this study, an analyte solution containing a specific probe labeled with FITC and a primer labeled with biotin was developed, and the sample was mixed with the analyte solution where the test strip was placed. The labeled analyte binds to the gold-labeled FITC-specific antibodies in the sample application area of the strip. Driven by capillary forces, travel through the membrane. On the one hand, only analyte-captured gold particles bound to immobilized biotin-ligand molecules as they passed through the line, generating a red-blue band over time. On the other hand, unbound gold particles migrated over the control band, where they were captured by species-specific antibodies. The mechanism of the HybriDetect universal lateral flow dipstick is presented Figure 4 (https://www.milenia-biotec.com/en/product/hybridetect/#nav-protocol) (accessed on 26 October 2022). For the detection method we developed for VZV—and as in any other tests that make use of the LF system for pathogen detection—the occurrence of a false-positive line on the dipstick is inevitable. Several factors contribute to these false positives, such as false amplification due to contamination, as well as the use of incompatible primers and/or probes that can result in unexpected false-positive signals [41,42,43,44,45,46]. As in this study, we recommend incubating the dipstick in the buffer solution for no longer than 5 min to avoid false-positive results. Incubation for times longer than 5 min may trigger the appearance of a false-positive line on the dipstick.

VZV is characterized by neurotropism and lifelong latent infection [47,48,49]. After primary infection, the virus gains access to neurons, establishes latency in the ganglia of the peripheral nervous system (PNS), and may later on reactivate and cause shingles [47,48,49,50,51]. The gene of interest for the RAA-LF system is the ORF63, which synthesizes a protein (IE63) that is expressed during both lytic and latent phases of infection [32]. The gene-encoded protein IE63 is located in the nucleus during lytic replication, and localizes to the cytoplasm of neurons during latency. However, in case of reactivation, the protein is present in both the nucleus and cytoplasm [52]. Although ORF63 is the most abundantly expressed gene at the levels of both protein and mRNA during latency [53,54], this RAA-LF system cannot be used to detect the latent form of VZV. Latent VZV infections are predominantly, if not exclusively, contained in the ganglia of the PNS [50,51,52], but the samples used for testing in this study are from skin lesions. Due to the mechanism of VZV latency, a positive test strip result from skin samples (not containing ganglia) would only be possible in the case of an active infection, either as a primary infection (chickenpox) or reactivation of the virus from latency as shingles [49,55].

The detection method developed for VZV in this study can be used as a point-of-care-test (POCT), especially in rural areas, because it can be easily done out of the laboratory by non-specialists. PCR may be the “gold standard” for laboratory diagnostics today, but RAA can be a useful complement and a potential substitute to this method, given its convenience and accessibility. In any case, timely detection of the causative agent is essential for infection control. Thus, having an easy, cheap, and handy detection test is advantageous in curbing possible VZV and/or MPXV outbreaks.

## 5. Conclusions

There is a constant need for laboratory diagnostics to determine the causative agent of a disease, and to be able to execute appropriate actions in a timely and fruitful manner. Making an accurate diagnosis for VZV infections can be difficult based on clinical manifestations alone, and, due to high VZV transmissibility, there is a vast need to develop a rapid detection method for this virus. Laboratory testing is important for detecting VZV, and PCR is the preferred diagnostic test given its accuracy and sensitivity. However, it is time-consuming and costly, and during outbreaks, timeliness is crucial. Therefore, the successful development of a convenient detection method for VZV greatly benefits the less developed areas of the world, in particular, as it saves both time and resources. In addition, it is also a potentially commercially viable product, thereby conferring great economic importance. Finally, given their convenience, rapid detection systems using RAA are likely to become the standard diagnostic method, ultimately replacing PCR, especially in regions where laboratory testing is not readily available.

## Figures and Tables

**Table 1:** Details of the collected varicella-zoster virus (VZV) samples.

Sample	Year	Location	Type	Age ^1^	Gender ^2^
1	2018	Rafaï	crust	29 y.o.	M
2		Rafaï	crust	8 m.o.	M
3		Bangui	crust	34 y.o.	M
4		Bangui	pus	13 y.o.	M
5		Bangassou	crust	unspecified	M
6		Bangassou	crust	unspecified	unspecified
7		Bangassou	crust	unspecified	F
8		Bangassou	crust	unspecified	F
9		Bangui	pus	41 y.o.	M
10	2019	Bimbo	crust	45 y.o.	M
11		unspecified	crust	21 y.o.	F
12		Bangui	crust	3 m.o.	M
13		Mbaïki	pus	28 y.o.	M
14		Bria	pus	36 y.o.	M
15	2020	Boda	crust	11 y.o.	F
16		Mbaïki	pus	27 y.o.	M
17		Nola	crust	45 y.o.	M
18	2021	Bimbo	crust	5 y.o.	F
19		Bimbo	crust	5 y.o.	M
20		Bimbo	crust	10 y.o.	M

^1^ Age: y.o.—years old; m.o.—months old; ^2^ Gender: M—male; F—female.

**Table 2:** Primers and probes to detect VZV using quantitative polymerase chain reaction (qPCR) and recombinase-aided amplification-lateral flow (RAA-LF) assays.

Name	Sequence (5′–3′)
qPCR F	CGCGTTTTGTACTCCGGG
qPCR R	CGGTTGATGTCCTCAACGAG
qPCR P	FAM-TGGGAGATCCACCCGGCCAG-TAMRA
RAA-LF F1	GATGTTAACGGAAAGATGGAATATGGATCTGC
RAA-LF R1	Biotin-CGACCCATTAGATAAAAGTCGAGGCATATG
RAA-LF P1	FAM-GTACTCCGGGTTGGGAGATCCACCCGGCCAGGCTC /idSp/GTTGAGGACATCAACCG-C3Sp

**Table 3:** Results of RT-PCR and RAA-LF tests using clinical samples.

Method	Positive	Negative	Total	PPV ^1^	NPV ^2^
RT-PCR	19	1	20	1	1
RAA-LF	19	1	20	1	1

^1^ PPV—positive predictive value; ^2^ NPV—negative predictive value.

## Data Availability

Not applicable.

## Supplementary Materials

The following supporting information can be downloaded at: https://www.mdpi.com/article/10.3390/diagnostics12122957/s1, Figure S1: RAA primer screening by PCR.
