# Supplementary material for: Rapid Detection of the Varicella-Zoster Virus Using a Recombinase-Aided Amplification-Lateral Flow System
Source: Diagnostics (Basel). 2022 Nov 25;12(12):2957. doi: 10.3390/diagnostics12122957 (PMC9777233; doi:10.3390/diagnostics12122957)
Supplement: Supplementary file 1 [file diagnostics-12-02957-s001.zip › diagnostics-1963415-supplementary.pdf]

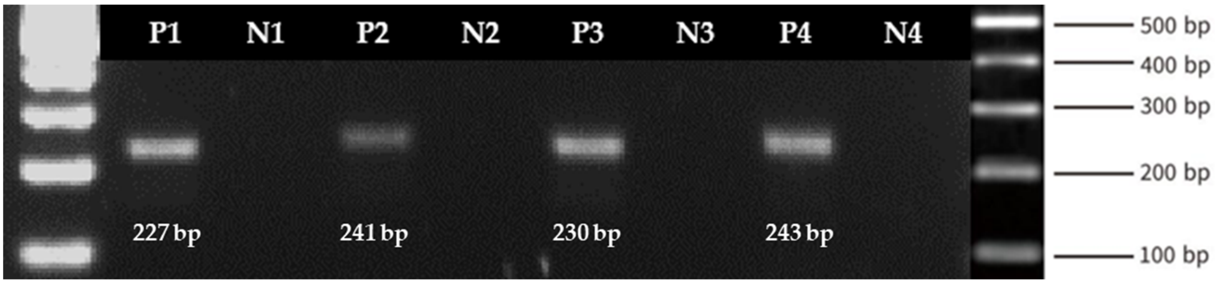

Figure S1. RAA primer screening by PCR. Four sets of primers were validated by polymerase chain reaction (PCR) to determine the amplification feasibility of the primers. The preferred primer pair was RAA-LF F1 and RAA-LF R1 (P1).

P – Primer; N – Negative control
